# Factors Associated with Influenza Vaccination of Hospitalized Elderly Patients in Spain

**DOI:** 10.1371/journal.pone.0147931

**Published:** 2016-01-29

**Authors:** Àngela Domínguez, Núria Soldevila, Diana Toledo, Pere Godoy, Jesús Castilla, Lluís Force, María Morales, José María Mayoral, Mikel Egurrola, Sonia Tamames, Vicente Martín, Jenaro Astray, Working Group of the Project PI12/02079

**Affiliations:** 1 Departament de Salut Pública, Universitat de Barcelona, Barcelona, Spain; 2 CIBER Epidemiología y Salud Pública (CIBERESP), Madrid, Spain; 3 Agència de Salut Pública de Catalunya, Barcelona, Spain; 4 Institut de Recerca Biomèdica de Lleida, Universitat de Lleida, Lleida, Spain; 5 Instituto de Salud Pública, Instituto de Investigación Sanitaria de Navarra, Pamplona, Spain; 6 Hospital de Mataró, Mataró, Spain; 7 Universitat de Valencia, Valencia, Spain; 8 Servicio de Vigilancia de Andalucía, Sevilla, Spain; 9 Hospital de Galdakao, Usansolo, Spain; 10 Dirección General de Salud Pública, Investigación, Desarrollo e Innovación, Junta de Castilla y León, León, Spain; 11 Universidad de León, León, Spain; 12 Consejería de Sanidad, Madrid, Spain; UNIFESP Federal University of São Paulo, BRAZIL

## Abstract

Vaccination of the elderly is an important factor in limiting the impact of influenza in the community. The aim of this study was to investigate the factors associated with influenza vaccination coverage in hospitalized patients aged ≥65 years hospitalized due to causes unrelated to influenza in Spain. We carried out a cross-sectional study. Bivariate analysis was performed comparing vaccinated and unvaccinated patients, taking in to account sociodemographic variables and medical risk conditions. Multivariate analysis was performed using multilevel regression models. We included 1038 patients: 602 (58%) had received the influenza vaccine in the 2013–14 season. Three or more general practitioner visits (OR = 1.61; 95% CI 1.19–2.18); influenza vaccination in any of the 3 previous seasons (OR = 13.57; 95% CI 9.45–19.48); and 23-valent pneumococcal polysaccharide vaccination (OR = 1.97; 95% CI 1.38–2.80) were associated with receiving the influenza vaccine. Vaccination coverage of hospitalized elderly people is low in Spain and some predisposing characteristics influence vaccination coverage. Healthcare workers should take these characteristics into account and be encouraged to proactively propose influenza vaccination to all patients aged ≥65 years.

## Introduction

During seasonal epidemics, large numbers of influenza infections may occur in all age groups. In most persons, influenza is a self-limiting illness, but serious secondary complications develop in some infected persons. Influenza affects global health and economies and the resulting illnesses often require hospitalization, overwhelming hospital and other health care systems and resulting in excess influenza-related deaths [1].

In elderly people, influenza causes complications of other underlying diseases. Increases in the annual estimated number of deaths resulting from influenza since the 1970s have been attributed to the disproportionate growth of the population aged ≥65 years, who account for 90% of deaths caused by influenza [2].

The capacity of influenza A and B viruses to undergo gradual antigenic change in their surface antigens is taken into account in seasonal influenza vaccination. The annual administration of seasonal influenza vaccine, especially in persons known to be at high risk of serious complications as a result of influenza infection, is the focus of current efforts to reduce the disease impact [1,2].

Observational studies in different communities have shown that annual influenza vaccination reduces hospitalizations and all-cause deaths in the elderly [3–6]. The World Health Organization strategy and action plan for healthy ageing in Europe, 2012–2020 considered the vaccination of older people in order to reduce the health risks (morbidity and mortality) as a priority intervention [7].

A systematic review of studies on the social determinants of seasonal influenza vaccination in adults aged ≥65 years suggests that the probability of receiving the vaccine is influenced by structural and healthcare-related social determinants [8].

In Spain, 17.6% of the population was aged ≥65 years in 2013 [9] and this is forecast to increase to 30.8% in 2050 [10]. The proportion of institutionalized persons in this age group is 3.6% [11].

The aim of this study was to investigate the factors associated with influenza vaccination coverage in people aged ≥65 years hospitalized due to causes unrelated to influenza in Spain.

## Materials and Methods

### Study design

We carried out a cross-sectional study in hospitalized patients aged ≥65 years from 19 hospitals located in the main cities of seven Spanish regions (Andalusia, Basque Country, Catalonia, Castile and Leon, Madrid, Navarra and Valencia Community) with unplanned hospital admission (patients not admitted for scheduled surgery or other treatments) due to causes other than acute respiratory disease, pneumonia or influenza-like illness were recruited between November 2013 and May 2014.

### Selection of patients

Patients included in the study were sought from patients admitted to the internal medicine service through the emergency department, and from patients admitted to the general surgery, otorhinolaryngology, ophthalmology, dermatology and traumatology services. Patients who did not give written consent were excluded, as were residents of nursing homes. In Spain, influenza vaccination is offered on site to all residents of nursing homes, in whom vaccine coverages are close to 100%, while suboptimal coverages are concentrated in non-institutionalized subjects who must attend to a health care centre to be vaccinated.

### Dependent and independent variables

The dependent variable was influenza vaccination in the 2013–2014 season. Patients were considered vaccinated with the seasonal influenza vaccine if they had received a dose of the vaccine during the 2013–2014 season. Information on the vaccination status was obtained from vaccination registers, hospital medical records, vaccination cards or primary healthcare records.

Specifically-trained health professionals used a structured questionnaire to collect information on independent variables by patient interview and review of medical records. Independent variables were grouped in three categories according to the socio-behavioural model proposed by other authors [12–14], predisposing characteristics, enabling resources and risk medical conditions. The following predisposing characteristics were recorded: age, sex, educational level, smoking, alcohol intake, vaccination status against pneumococcal disease and influenza vaccination history in the three previous influenza seasons. Variables related to social support were collected to measure enabling resources: marital status, number of general practitioner (GP) visits during the last year, number of hospital visits during the last year, whether the patient lived alone or at home with cohabitants, and the Barthel index, which has a total score ranging from 0 (complete dependence) to 100 (complete independence), as a measurement of limitations in activity in the patients included in the study. Risk medical conditions included were those frequently associated with recommendations on influenza vaccination [1,2]: chronic obstructive pulmonary disease, chronic respiratory failure, history of pneumonia during the last two years, neoplasia, transplantation, immunosuppressive treatment, asplenia, diabetes, renal failure, nephrotic syndrome, autoimmune disease, AIDS, asymptomatic HIV infection, congestive heart disease, disabling neurological disease, obesity, chronic liver disease, hemoglobinopathy or anaemia, cognitive dysfunction, convulsions and neuromuscular disease.

### Statistical analysis

A bivariate analysis was made to compare vaccinated and unvaccinated patients taking into account the sociodemographic variables and risk medical conditions. As each Spanish region may introduce specific vaccination programs for specific population groups and because regions have some degree of autonomy in organizing health services, persons living in the same region tend to have similar access to health care. Therefore, to estimate the crude and adjusted odds ratio (OR), we used multilevel regression models that consider the outcome variable among people from the same region, in order to obtain accurate statistical estimates of vaccination predictors [15]. Covariates were introduced into the model using a backward stepwise procedure, with a cut-off point of p <0.2.

Model 1 included only variables related to enabling resources; model 2 also included predisposing variables and model 3 included enabling resources, predisposing variables and risk medical conditions.

The analysis was performed using the SPSS v.18 statistical package and the R v3.1.2 statistical software (http://cran.r-project.org).

### Ethical considerations

All data collected were treated as confidential, in strict observance of legislation on observational studies. The study was approved by the Ethics Committees of the hospitals involved (Comité Ético de Investigación Clínica del Hospital Clínic de Barcelona; Comité Ético de Investigación Clínica del Hospital Universitari Mutua de Terrassa; Comité Ético de Investigación Clínica de la Corporació Sanitaria Parc Taulí de Sabadell; Comité Ético de Investigación Clínica del Hospital de Mataró, Consorci Sanitari del Maresme; Comité Ètic d’Investigació Clínica de la Fundació Unio Catalana Hospitals; Comité Ético de Investigación Clínica Área de Euskadi; Comité Ético de Investigación Clínica Área de Salud de Burgos y Soria; Comité Ético de Investigación Clínica Área de Salud de León; Comité Ético de Investigación Clínica Área de Salud Valladolid– Este; Comité Coordinador de Ética de la Investigación Biomédica de Andalucía; Comité Ético de Investigación Clínica del Hospital Ramón y Cajal, Madrid and Comité Ético de Investigación Clínica del Consorcio Hospital General Universitario de Valencia). Written informed consent was obtained from all patients included in the study.

## Results

Of the 1038 patients included in the study, 602 (58%) had received the influenza vaccine in the 2013–2014 season.

The distribution of the study variables (predisposing characteristics, enabling resources and medical risk conditions) in vaccinated and unvaccinated patients is shown in Table 1. According to age, patients aged ≥90 years had the highest vaccination coverage (64%), followed by the 85–89 years age group (63.7%), and the lowest coverage was observed in the 65–69 years age group (44.2%). A history of influenza vaccination in any of the three previous seasons was found in 78.3% of persons vaccinated during the 2013–14 season compared with only 21.7% in unvaccinated patients. Single patients had lower vaccination rates than patients with partners (41.9% and 61.3%, respectively). Patients who made ≥3 GP visits in the previous year had higher vaccination rates than those who did not (62.2% and 37.8%, respectively). Patients living at home in cohabitation had higher rates (60.0%) than those living alone (49.2%).

**Table 1:** Distribution of vaccinated and non-vaccinated patients according to sociodemographic characteristics, clinical conditions and history of vaccination.

	Vaccinated patients n (%), N = 602	Unvaccinated patients n (%), N = 436	Crude OR	p value
**Predisposing characteristics**				
**Age group**				
65–69	69 (44.2%)	87 (55.8%)	1	
70–74	112 (57.1%)	84 (42.9%)	1.69 (1.10–2.59)	0.02
75–79	144 (58.1%)	104 (41.9%)	1.71 (1.14–2.56)	0.01
80–84	166 (62.9%)	98 (37.1%)	2.11 (1.41–3.16)	<0.001
85–89	79 (63.7%)	45 (36.3%)	2.08 (1.27–3.42)	0.003
≥90	32 (64.0%)	18 (36.0%)	2.08 (1.06–4.08)	0.03
**Sex**				
Male	337 (60.4%)	221 (39.6%)	1	
Female	265 (55.2%)	215 (44.8%)	0.78 (0.61–1.00)	0.05
**Education level**				
No/primary education	470 (58.2%)	337 (41.8%)	1	
Secondary or higher	127 (56.2%)	99 (43.8%)	0.84 (0.61–1.16)	0.29
**Smoking status**				
Non smoker	336 (59.1%)	233 (40.9%)	1	
Ex-smoker	224 (57.4%)	166 (42.6%)	0.98 (0.75–1.28)	0.87
Smoker	40 (51.9%)	37 (48.1%)	0.79 (0.48–1.28)	0.33
**Alcohol intake**				
No	582 (58.3%)	417 (41.7%)	1	
Yes	17 (51.5%)	16 (48.5%)	0.74 (0.37–1.49)	0.40
**23-valent pneumococcal polysaccharide vaccine**				
No	274 (44.0%)	349 (56.0%)	1	
Yes	328 (79.0%)	87 (21.0%)	6.18 (4.39–8.69)	<0.001
**Influenza vaccine in any of the 3 previous seasons**				
No	62 (17.8%)	286 (82.2%)	1	
Yes	540 (78.3%)	150 (21.7%)	16.76 (12.03–23.34)	<0.001
**Enabling resources**				
**Marital status**				
Married/Cohabiting	356 (61.3%)	225 (38.7%)	1	
Single	36 (41.9%)	50 (58.1%)	0.42 (0.26–0.67)	0.001
Widowed	195 (57.4%)	145 (42.6%)	0.85 (0.65–1.12)	0.26
Separated/Divorced	12 (46.2%)	14 (53.8%)	0.57 (0.26–1.26)	0.16
**No. of GP visits**				
0–2	160 (48.5%)	170 (51.5%)	1	
≥3	435 (62.2%)	264 (37.8%)	1.72 (1.31–2.25)	<0.001
**No. of hospital visits**				
0–2	358 (56.5%)	276 (43.5%)	1	
≥3	236 (60.1%)	157 (39.9%)	1.17 (0.90–1.51)	0.24
**Household size**				
Live alone	97 (49.2%)	100 (50.8%)	1	
Lives with cohabitant	503 (60.0%)	336 (40.0%)	1.52 (1.11–2.08)	0.01
**Barthel index**				
≥40	537 (58.9%)	374 (41.1%)	1	
< 40	63 (50.4%)	62 (49.6%)	0.73 (0.50–1.07)	0.11
**Risk medical conditions**				
No	69 (59.5%)	47 (40.5%)	1	
Yes	533 (57.8%)	389 (42.2%)	0.95 (0.64–1.41)	0.80

OR: odds ratio, GP: general practitioner

No differences were observed in vaccinated and unvaccinated patients with respect to educational level, sex, level of dependency or the presence of risk medical conditions. S1 Table shows the distribution of risk medical conditions in vaccinated and unvaccinated patients.

The results of the multilevel regression model are shown in Table 2. Variables related to enabling resources (Model 1) significantly associated with vaccine uptake were being single (OR = 0.55; 95% CI 0.32–0.93), ≥3 GP visits in the previous year (OR = 1.72; 95% CI 1.31–2.26) and a Barthel index <40 (OR = 0.65; 95% CI 0.44–0.97).

**Table 2:** Results of multilevel regression for estimated of factors associated with influenza vaccine.

	Model 1 (*Enabling resources*) Adjusted OR (95%CI)	p value	Model 2 (*Model 1 + Predisposing characteristics*) Adjusted OR (95%CI)	p value	Model 3 (*Model 2 + Risk medical conditions*) Adjusted OR (95%CI)	p value
**Enabling resources**						
**Marital status**						
Married/Cohabiting	1		1		1	
Single	0.55 (0.32–0.93)	0.03	0.55 (0.31–0.98)	0.04	0.56 (0.32–0.98)	0.04
Widowed	1.00 (0.72–1.38)	0.98	1.10 (0.77–1.58)	0.60	1.11 (0.77–1.59)	0.57
Separated/Divorced	0.72 (0.31–1.68)	0.45	0.87 (0.36–2.11)	0.76	0.85 (0.35–2.06)	0.72
**No. of GP visits**						
0–2	1		1		1	
≥3	1.72 (1.31–2.26)	<0.001	1.57 (1.16–2.11)	0.003	1.61 (1.19–2.18)	0.002
**Household size**						
Live alone	1		1		1	
Lives with cohabitant	1.35 (0.91–2.01)	0.13	1.35 (0.88–2.07)	0.17	1.36 (0.89–2.09)	0.16
**Barthel index**						
≥40	1		1		1	
< 40	0.65 (0.44–0.97)	0.03	0.71 (0.46–1.08)	0.11	0.72 (0.47–1.09)	0.12
**Predisposing characteristics**						
**Sex**						
Male			1		1	
Female			0.77 (0.55–1.10)	0.14	0.76 (0.53–1.08)	0.12
**Alcohol intake**						
No			1		1	
Yes			0.48 (0.20–1.12)	0.09	0.49 (0.21–1.17)	0.11
**Influenza vaccine in any of the 3 previous seasons**						
No			1		1	
Yes			13.07 (9.14–18.67)	<0.001	13.57 (9.45–19.48)	<0.001
**23-valent pneumococcal polysaccharide vaccine**						
No			1		1	
Yes			1.95 (1.37–2.78)	<0.001	1.97 (1.38–2.80)	<0.001
**Risk medical conditions**						
No					1	
Yes					0.73 (0.47–1.14)	0.17

OR: odds ratio, GP: general practitioner

Predisposing characteristics (Model 2) associated with influenza vaccination in the 2013–14 season were influenza vaccination in any of the 3 previous seasons (OR = 13.07; 95% CI 9.14–18.67) and 23-valent pneumococcal polysaccharide vaccination (OR = 1.95; 95% CI 1.37–2.78). Single status and ≥3 GP visits were also associated with influenza vaccination.

After the inclusion of risk medical conditions (Model 3), the variables associated were the same as in model 2, with only very-slight increases in the values of the OR for ≥3 GP visits (OR = 1.61; 95% CI 1.19–2.18); for influenza vaccination in any of the 3 previous seasons (OR = 13.57; 95% CI 9.45–19.48); and for previous vaccination with the 23-valent pneumococcal polysaccharide vaccine (OR = 1.97; 95% CI 1.38–2.80).

## Discussion

The results of this study show that influenza vaccination coverage in elderly hospitalized patients in Spain (58.0%) is clearly lower than the 75% target proposed by the World Health Assembly for people aged ≥65 years [16]. A Council’s recommendation of the Commission of the European Community states that concerted action at the community level should be taken to contain seasonal influenza by encouraging vaccination among at-risk groups, with the purpose of reaching the target of 75% vaccination coverage of the older age groups recommended by the WHO, as far as possible by 2015 [17]. This target was not reached, with the median being 44.7% and only two member states achieving the target in the 2012–13 season: the Netherlands and the United Kingdom [18].

The coverage obtained in this study is very close to that of other Spanish and international studies. In women in the same age group in Galicia, vaccination coverage between 2000 and 2004 was 56.8% [19]. In a more recent study in Navarra [20], the coverage in non-institutionalised persons aged ≥65 years in the 2010–11 season was 58.6% and, similar to the results of the present study, the highest coverage was in the 85–89 years age group. In Israel [21], the coverage in persons aged ≥65 years in 2008–09 was 59.2%, and an increase was also observed in older age groups.

Other authors have found higher coverages. In the United States in the 2013–14 influenza season, the coverage was 64.7% in persons aged ≥65 years [22]. The coverage was 73% in 2010–11 in patients aged >65 years seen by general practitioners included in the computerized disease-surveillance system in France [23]. In many European countries, the vaccine coverage in people aged >65 years is even lower than was found in our study [18,24].

In all the models in the present study, single marital status was associated with a lower coverage and ≥3 GP visits in the previous year with a higher coverage. Single marital status was also associated with a lower coverage in an Italian study [14]. In an Irish study [25] widowed persons had a lower coverage than married ones, but the Spanish study [19] found that older married women had a significantly-higher probability of being vaccinated.

The association between vaccination coverage and the number of GP visits is in agreement with other studies in Spain and other countries. [19,20,25–30]

Like most studies [14,22,25,27,30,31], no association was found between sex and influenza vaccination coverage. However, studies in Spain and Israel have found higher coverages in males [20,21,26]. We found no association between vaccination coverage and educational level, in agreement with other authors [19,21,25,27,32].

An association between a higher Barthel index and vaccination coverage was found in model 1, but was not exhibited in models 2 and 3. There is contradictory evidence on the relationship between influenza vaccination and the level of dependence. A Hong Kong study by Lau et al [31] found that participants with a higher Barthel index were more likely to be vaccinated, but in the final multivariable logistic model no association was found. In contrast, a study in Navarra found that a high level of dependence was associated with vaccination [20].

In models 2 and 3, we found a very close association between influenza vaccination in any of the three previous seasons and influenza vaccination in 2013–14, in agreement with other studies in Europe [20,23,27], Canada [29] and China [31]. Likewise, a history of pneumococcal vaccination was associated with influenza vaccination. A US study [33] found that 95% of persons vaccinated against influenza had received the pneumococcal vaccine. In our study, the figure was 54.5%, but in models 2 and 3 this variable was associated with influenza vaccination. Two studies in Canada [29,30] found that participants reporting having received influenza vaccination at least once were significantly more likely to report having received the pneumococcal vaccine.

We found no association between risk medical conditions and vaccination coverage, similar to the results of a Brazilian study [32]. Lau et al [31] found that patients with more risk medical conditions were less likely to be vaccinated, but other authors have found contrary results [14,20,23].

The role of primary care physicians in promoting vaccination in the elderly has been widely recognized [23,34,35]. In a recent Spanish study [36], influenza vaccination of patients aged ≥65 years was associated with their physicians having favourable opinions about vaccination. Therefore, the promotion of vaccination of primary care physicians by improving their opinions and attitudes about influenza vaccination and taking into account scientific evidence may have a beneficial effect on coverages in their patients. Some authors [37,38] have pointed out that healthier persons are more likely to receive the vaccine than less-healthy subjects (health vaccine bias) and if observational studies do not control adequately for those differences, the benefits of influenza vaccination might be exaggerated. One way to avoid this kind of bias, could be the design of appropriately-powered RCTs [30,39]. However, RCT are unacceptable among groups already advised to receive the vaccine annually [2] and observational studies using bias-reducing methods likely represent the only possible option to assess the effectiveness of the influenza vaccine in the elderly [40]. In a study carried out during 10 seasons with no evidence of a healthy-vaccine bias, the effectiveness of the vaccine against hospitalization was demonstrated [4]. A review of 75 studies in the elderly (only one RCT, with the rest being cohort and case-control studies) concluded that the public health safety profile of the vaccine appears to be acceptable, but the low quality of the available evidence precluded clear conclusions being reached on the effects of the vaccine in the elderly [41]. Osterholm et al. stated that evidence of protection for the present generation of influenza vaccines in adults aged ≥65 years is lacking and that new vaccines based on novel antigens are needed; however the authors agreed that, in the meantime, public support for present vaccines are the best intervention available for seasonal influenza and should be maintained [42].

The World Health Organization (WHO), after considering that the available evidence suggests that influenza vaccines are less effective in people aged ≥65 years than in young adults, states that vaccination is still the most efficacious public health tool for the protection of elderly individuals against influenza and encourages its use in elderly persons because they have the highest risk of mortality from the complications of influenza [43].

Advertising, provider and patient mailing, registry-based telephone calls and patient education could also increase vaccination rates [44].

This study, like all observational studies has strengths and limitations. The main strength is that the vaccination status was obtained from written documents (hospital medical records, vaccination cards or primary healthcare registers) and, therefore, it is unlikely that this information was biased. With the exception of two studies [20,30], in all the other studies cited information on vaccination was obtained directly from the patient or physician. Another strength is that only patients with unplanned hospital admission were included. These patients enter for a variety of reasons and tend to be more representative of the general population. A possible limitation might be that we excluded patients admitted to hospital for respiratory disease, pneumonia or influenza-like illness. Patients hospitalized due to these diseases had probably received the influenza vaccine less frequently than the overall population of elderly people.

Another limitation is that other, unaccounted-for factors might explain some of the relationships observed. In the multilevel regression analysis we took into account several variables that were not included in the final models. Therefore, the associations that were found in these final models might be considered in future strategies to improve influenza vaccination coverages in the elderly. Another possible limitation is that no standard definitions of predisposing characteristics or enabling resources are available. We followed criteria similar to those used by other authors [12–14] and, therefore the comparison may be considered valid. Finally, because this is a cross-sectional study, no causal relationship can be established. However, the study identified some variables that are associated with vaccination coverage in hospitalized patients and could help improve vaccination strategies in the elderly.

In conclusion, the results of this study show that influenza vaccination coverages in elderly hospitalized patients are low in Spain and should be increased. Some predisposing characteristics were associated with a higher influenza vaccination coverage. Healthcare workers should take these characteristics into account and be encouraged to proactively propose influenza vaccination to all patients aged ≥65 years.

## Supporting Information

S1 Table Distribution of vaccinated and non-vaccinated patients according to risk medical conditions. (DOCX)
