# Supplementary material for: Factors Associated with Influenza Vaccination of Hospitalized Elderly Patients in Spain
Source: PLoS One. 2016 Jan 29;11(1):e0147931. doi: 10.1371/journal.pone.0147931 (PMC4732680; doi:10.1371/journal.pone.0147931)
Supplement: S1 Table — (DOCX) [file pone.0147931.s001.docx]

**S1 Table. Distribution of vaccinated and non-vaccinated patients according to risk medical conditions**

|  | **Vaccinated patients n (%), N=602** | **Unvaccinated patients n (%), N=602** | **Crude OR** | **p value** |
| --- | --- | --- | --- | --- |
| **Chronic obstructive pulmonary disease** |  |  |  |  |
| Yes | 83 (60.1%) | 55 (39.9%) | 1.17 (0.80 – 1.69) | 0.42 |
| No | 517 (57.8%) | 377 (42.2%) | 1 |  |
| **Chronic respiratory failure** |  |  |  |  |
| Yes | 73 (57.0%) | 55 (43.0%) | 1.02 (0.69 – 1.51) | 0.91 |
| No | 522 (58.3%) | 373 (41.7%) | 1 |  |
| **Pneumonia during the last two years** |  |  |  |  |
| Yes | 26 (57.8%) | 19 (42.2%) | 0.96 (0.52 – 1.76) | 0.89 |
| No | 566 (57.8%) | 413 (42.2%) | 1 |  |
| **Neoplasia** |  |  |  |  |
| Yes | 121 (55.3%) | 98 (44.7%) | 0.82 (0.60 – 1.11) | 0.20 |
| No | 480 (58.8%) | 337 (41.2%) | 1 |  |
| **Transplantation** |  |  |  |  |
| Yes | 4 (80.0%) | 1 (20.0%) | 2.58 (0.28 – 23.32) | 0.40 |
| No | 597 (57.9%) | 434 (42.1%) | 1 |  |
| **Immunosuppressive treatment** |  |  |  |  |
| Yes | 18 (50.0%) | 18 (50.0%) | 0.69 (0.35 – 1.35) | 0.27 |
| No | 580 (58.2%) | 417 (41.8%) | 1 |  |
| **Asplenia** |  |  |  |  |
| Yes | 3 (60.0%) | 2 (40.0%) | 1.02 (0.17 – 6.18) | 0.98 |
| No | 594 (58.5%) | 421 (41.5%) | 1 |  |
| **Diabetes** |  |  |  |  |
| Yes | 226 (59.5%) | 154 (40.5%) | 1.15 (0.88 – 1.49) | 0.30 |
| No | 376 (57.1%) | 282 (42.9%) | 1 |  |
| **Renal failure** |  |  |  |  |
| Yes | 139 (64.4%) | 77 (35.6%) | 1.41 (1.03 – 1.93) | 0.03 |
| No | 462 (56.3%) | 358 (43.7%) | 1 |  |
| **Nephrotic syndrome** |  |  |  |  |
| Yes | 3 (60.0%) | 2 (40.0%) | 0.97 (0.16 – 5.89) | 0.97 |
| No | 597 (58.0%) | 433 (42.0%) | 1 |  |
| **Congestive heart disease** |  |  |  |  |
| Yes | 224 (62.6%) | 134 (37.4%) | 1.55 (1.17 – 2.07) | 0.003 |
| No | 376 (55.7%) | 299 (44.3%) | 1 |  |
| **Chronic liver disease** |  |  |  |  |
| Yes | 33 (60.0%) | 22 (40.0%) | 1.15 (0.66 – 2.02) | 0.62 |
| No | 567 (58.0%) | 410 (42.0%) | 1 |  |
| **Neuromuscular disease** |  |  |  |  |
| Yes | 14 (48.3%) | 15 (51.7%) | 0.64 (0.31 – 1.35) | 0.25 |
| No | 586 (58.4%) | 417 (41.6%) | 1 |  |

OR: Odds Ratio
